# TGF-β Is Critical for Ovarian Cancer Migration, Invasion, and Chemosensitivity

**DOI:** 10.3390/cancers18142268

**Published:** 2026-07-15

**Authors:** Christopher Elms, Wei Wei, Michael J. Birrer

**Affiliations:** 1 Department of Biochemistry and Molecular Biology, University of Arkansas for Medical Sciences, Little Rock, AR 72202, USA; ctelms@uams.edu; 2 Biotherapeutics Pharmaceutical Science, Pfizer Inc., New York, NY 10001, USA; falcon.wei@gmail.com

**Keywords:** TGF-β, ovarian cancer, chemotherapy resistance, cytoreductive surgery, tumor microenvironment

## Abstract

**Background/Objectives:** Standard treatment for ovarian cancer includes surgery and chemotherapy, either as primary “debulking” surgery followed by adjuvant chemotherapy or neoadjuvant chemotherapy followed by interval debulking surgery. The degree of surgical resection correlates with overall survival, with “suboptimal debulking” (visible remaining cancer) being a poor prognostic variable. TGF-β pathway activation is associated with ovarian cancers that cannot be optimally debulked. To test the role of this pathway in ovarian cancer biology, modulation of the pathway was performed in in vitro and in vivo ovarian cancer model systems. **Methods:** A small molecule TGF-β receptor 1 (TGFBR1) kinase inhibitor, LY2157299, was used to test the in vitro cellular adhesion, proliferation, migration and invasive ability of ovarian cancer cells in culture. A TGF-β responsive reporter construct was used to determine the role of TGF-β pathway in those interactions. To test the effects of TGF-β inhibition in vivo, TGFBR1 and TGFBR2 CRISPR knockout cells were generated and examined for growth and sensitivity to chemotherapy. **Results:** For ovarian cancer cells responsive to exogenous TGF-β1 treatment, LY2157299-mediated the TGF-β pathway inhibition decreased cell migration and invasion, as well as mesothelial cell to cancer cell adhesion, proliferation, and migration. TGFBR1 and TGFBR2 CRISPR knockout cells showed decreased proliferation baseline or when treated with TGF-β and LY2157299. TGFBR2 CRISPR cells also showed increased sensitivity to cisplatin, with a significant decrease in tumor weight in vivo. **Conclusions:** TGF-β pathway inhibition successfully targeted many of the cancer cell behaviors that could contribute to suboptimally debulked patients and showed an additive anti-tumor effect in conjunction with cisplatin. LY2157299 treatment is clinically limited due to solubility, but other TGF-β inhibitors could be highly efficacious treatment options.

## Simple Summary

Ovarian cancer (OC) ranks fifth overall in cancer deaths for women in the United States, and current treatment options frequently lead to recurrence of the disease. Our research has correlated activation of the transforming growth factor-β (TGF-β) pathway in ovarian cancer with cancer cell behaviors potentially linked to inadequate debulking surgery outcomes (>2 cm visible remaining cancer). We hypothesize that inhibition of the TGF-β pathway will inhibit ovarian cancer biologic characteristics consistent with those necessary to produce tumor spread throughout the abdomen in a fashion that predisposes to suboptimal debulking after surgery. Inhibition of the TGF-β pathway may provide a powerful new treatment approach for OC patients who would otherwise have unfavorable surgical outcomes.

## 1. Introduction

Epithelial ovarian cancer (EOC) is a gynecologic cancer with the highest case fatality rate and ranks fifth for overall cancer deaths among all age groups (4% of all cancer deaths in women) [1]. The estimated number of new ovarian cancer cases and deaths in 2025 are 20,890 and 12,730, respectively [1]. EOC makes up the majority (85%) of these cases, with the most common subtype being high-grade serous ovarian carcinoma (HGSOC). Most HGSOCs are diagnosed at an advanced stage (58%) due to largely non-specific symptoms and the absence of an early detection test [2]. The resulting late-stage diagnosis leads to a five-year survival rate of only 50.9% [3]. The standard treatment for ovarian cancer is cytoreductive or “debulking” surgery followed by platinum-based chemotherapy (an equally acceptable alternative is neoadjuvant therapy followed by interval debulking surgery) [4,5,6]. Resection of all visible disease is associated with favorable disease and treatment outcomes but often requires aggressive surgical strategies that can increase post-operative patient morbidity [7]. Patients who are suboptimally debulked are found to have a poor prognosis and lower response rates to chemotherapy [8]. This outcome certainly results in part from the quality of the surgical approach but also from the underlying biology of the tumor.

A meta-analysis of 1525 publicly available expression profiling assays on HGSOC identified a 200-gene expression signature of primary ovarian tumors that specifically predicted the outcome of debulking surgery [9]. The signature included multiple genes regulated by transforming growth factor-β (TGF-β), indicating activation of the pathway in suboptimally debulked ovarian cancer, which may be responsible for the aggressive behavior and invasiveness of these tumors [9,10,11]. The activation of the TGF-β pathway signals growth inhibitory effects on normal epithelial cells through downstream molecules such as Smad2 and Smad3, regulating differentiation, migration, and cell growth [12,13]. However, in cancer cells, it is reprogrammed in a paradoxical way to induce epithelial-mesenchymal transition (EMT) and tumor cell dissemination [14,15,16].

In this study, we evaluated the anti-tumor effect of TGF-β signaling inhibition on the cellular components of ovarian cancer in vitro and in vivo. Upon treatment with a small molecule TGF-β receptor 1 (TGFBR1) kinase inhibitor, LY2157299 (Eli Lilly), significant decreases were observed in ovarian cancer cell migration, invasion, and adhesion to mesothelial cells, as well as the interactions between cancer cells and tumor microenvironment components, including tumor-associated fibroblasts (TAFs). Along with these observations, TGF-β receptor knockout by CRISPR significantly inhibited the intraperitoneal tumorigenicity of SKOV3 cells in immunocompromised mice and further sensitized the tumor to cisplatin treatment at a dosage which had no significant effect on the control xenografts. Our work shows that TGF-β pathway inhibition could be a potential therapeutic strategy for neoadjuvant and combination therapies and improve the number of women with suboptimal surgical outcomes.

## 2. Materials and Methods

### 2.1. CAGA12-Luciferase Reporter Assay

Two immortalized ovarian epithelial cell lines (HOSE-80, HOSE-120) and 15 HGSOC cell lines (A1847, COV362, OV1063, OV90, OVCAR10, OVCAR2, OVCAR3, OVCAR4, OVCAR5, OVCAR8, OVSAHO, PEO-1, SKOV3, UCI107, UWB1.289) were tested with a CAGA12-Luciferase reporter assay for TGF-β responsiveness [17]. All cell lines had been utilized previously in the Birrer lab, and genetic information for each of these cell lines can be acquired from the ATCC STR database or the cBioPortal for Cancer Genomics. For each cell line tested, 8000 cells were plated in RPMI1640 + 10% FBS and incubated for 24 h on a 96-well plate. Co-transfection with 200 ng CAGA12-Luc + 20 ng R-Luc by Fugene HD was conducted and incubated for 6 h, after which the cells were treated with TGF-β (2 ng/mL) in RPMI1640+0.1% FBS, LY2157299 (5 µM), or both for 24 h. The Dual-Luciferase^®^ Reporter Assay (Promega, Madison, WI, USA, E1910) was conducted according to the manufacturer’s protocol in a white plate. The fold changes of normalized luciferase activity were calculated to determine if the cell line tested was TGF-β responsive, had baseline TGF-β activity, or was unresponsive to TGF-β. The cell lines were grouped according to their fold change into TGF-β responsive (inducible TGF-β activity only), baseline signaling (endogenous TGF-β activity and susceptible to LY2157299), and unresponsive (no response to TGF-β and no effect by LY2157299).

### 2.2. Ovarian Cancer Cell Migration and Invasion Assay

A Boyden chamber-based migration assay was conducted under blind conditions for SKOV3, OVCAR4, and OVCAR8 in a 24-well cell culture insert with an 8.0 µm pore size PET membrane (BD Biosciences, Franklin Lakes, NJ, USA). The cells were placed under a 0 to 0.5% FBS gradient for the SKOV3 cell line (25,000 cells in suspension per insert) or a 0 to 4% FBS gradient for OVCAR4 and OVCAR8 (50,000 and 35,000 cells, respectively, in suspension per insert) and treated with either TGF-β (2 ng/mL), LY2157299 (5 µM), or both. The differing FBS gradients were determined by titration of the responsiveness to FBS in each cell line during assay protocol generation. The reagents were added in the upper and lower chambers, and cells were allowed to migrate overnight. Migrated cells on the underside were fixed and stained with a Diff-Quick Stain Set (Dade Behring, Deerfield, IL, USA) and quantified by counting under 40× magnification. The relative fold change of migrated cells in each condition was measured to calculate the effect of LY2157299 on cancer cell migration.

The invasion assay for SKOV3 cells was performed in BD BioCoat™ Growth Factor Reduced Matrigel Invasion Chambers (Corning, Glendale, AZ, USA) with an 8.0 µm pore size PET membrane (50,000 cells per insert). The cells were treated with TGF-β (2 ng/mL), LY2157299 (5 µM), or both in the upper and lower chambers and incubated for 24 h. Chemotaxis was established via a 0 to 2% FBS gradient. Using the same quantification protocol, the relative fold change of invaded cells was calculated to determine the effect of LY2157299 on cancer cell invasion.

### 2.3. TGF-β Signaling Inhibition in Stromal Cells

TAFs (IHFOT208), generated through stably introducing human telomerase reverse transcriptase (hTERT) and subculturing for more than 30 passages, were cultured and then incubated with TGF-β (2 ng/mL), LY2157299 (5 µM), or both. Western blotting for p-Smad2 (S465) and Smad2 was conducted on samples taken at 1 and 24 h. Mesothelial cells (HM3), available from the Brigham and Women’s Hospital (BWH) Cell Culture Core, were cultured in M199/MCDB106 medium supplemented with 15% newborn calf serum, 10 ng/mL EGF, 0.4 μg/mL hydrocortisone, and 1% pen/strep. The cells were incubated with TGF-β (2 ng/mL), LY2157299 (5 µM), or both, similar to the IHFOT208 cells. Western blotting for p-Smad2 (S465) and Smad2 was then conducted to determine the inhibition of Smad2 phosphorylation, similar to the TAFs.

### 2.4. Western Blot

Total protein lysate was prepared from non-confluent cell culture by RIPA buffer with a cocktail of protease and phosphatase inhibitors (Roche, Indianapolis, IN, USA). Protein concentration was estimated with a BCA Protein Assay kit (Thermo Scientific, Waltham, MA, USA). In total, 20 µg of protein was separated by 4–20% SDS-PAGE (Bis-Tris gel, Invitrogen, Carlsbad, CA, USA) and transferred to a nitrocellulose membrane. Primary antibody incubation (4 °C, overnight) was performed with anti-phospho-Smad2 (S425/S427) (#3101) (Cell Signaling, Danvers, MA, USA), anti-Smad2 (#3103) (Cell Signaling,) or anti-TGFBR2 (ab184948) (Abcam, Waltham, MA, USA). Antibody detection was performed with horseradish peroxidase (HRP) conjugated antibodies and the SuperSignal Chemiluminescence Substrate system (Thermo Scientific, Waltham, MA, USA).

### 2.5. Cell Adhesion Assay

HM3 cells (BWH Cell Culture Core) were cultured according to the manufacturer’s instructions. To test TGF-β signaling blockage on ovarian cancer cell adhesion, mesothelial cells were plated overnight on a flat-bottom 96-well at 50,000 cells/well to establish an adherent monolayer. SKOV3 and OVCAR8 cells were pretreated with TGF-β (2 ng/mL) and LY2157299 (5 µM) in 1% FBS once every 24 h for 6 days and labeled with 1 μM of Calcein-AM for 30 min and washed twice with RPMI immediately before being added onto the mesothelial monolayer (10,000 cells/well). After incubation at 37 °C for the indicated time intervals, the plate was washed with PBS and vigorously shaken to remove unattached cells. The green fluorescence was quantified using a plate reader before and after shaking. The percentage of attached cells was calculated to indicate the adhesive activity. To test the impact of TGF-β signaling blockage in mesothelial cells on ovarian cancer cell adhesion, primed HM-3 cells were generated by culturing as previously stated and treated once every 24 h by 2 ng/mL TGF-β with and without LY2157299 (5 µM) for 4 days prior to conducting the assay. The primed mesothelial cells and routinely cultured SKOV3 or OVCAR8 cells were subjected to the adhesion assay, as described above.

### 2.6. Ovarian Cancer Cell and Fibroblasts Proliferation Assay

For the proliferation assay, SKOV3-Luc cells were generated via transfection of a Luciferase response element, and then 1000 cells/well were co-cultured with 1000 IHNOF303 (normal fibroblasts)/well in a 96-well plate in 1% FBS. The co-cultures were divided into 3 groups: (1) SKOV3-Luc cells cultured untreated; (2) SKOV3-Luc with IHNOF303; and (3) SKOV3-Luc with IHNOF303 + LY2157299. The cells were incubated for a total of 8 days, and cells were lysed on days 1, 3, 6, and 8. The Luciferase assay for SKOV3 proliferation was then conducted for each group. The fluorescence was then measured, and proliferation of the cells was calculated.

### 2.7. Quantitative PCR Analysis

Real-time quantitative PCR was performed on 50 ng of total RNA with a SuperScript^®^ III Platinum^®^ One-Step qRT-PCR Kit (Invitrogen, Carlsbad, CA, USA) in an iCycler iQ Real-Time PCR Detection System (Bio-Rad Laboratories, Hercules, CA, USA). The expression levels of housekeeping gene GAPDH were used as internal controls to normalize the amount of RNA input. The primer sequences are: MMP2-F: ATGCCGCCTTTAACTGGAG; MMP2-R: GGGAAGCCAGGATCCATTTT; PLAU-F: TGACCCACAGTGGAAAACAG; PLAU-R: CCAGCTCACAATTCCAGTCA; PAI-1-F: ACCGCAACGTGGTTTTCTCA; PAI-1-R: TTGAATCCCATAGCTGCTTGAAT; GAPDH-F: GAAGGTGAAGGTCGGAGTC; GAPDH-R: CATGTAGTTGAGGTCAATGAAGG. Annealing temperature for all primer pairs was set at 59 °C. Final primer concentration in the qRT-PCR reaction was set at 0.2 μM.

### 2.8. TGFBR1 or TGFBR2 Knockout by CRISPR

SKOV3 cells with inducible Cas9 overexpression were generated by infection with lentivirus particles generated from pCW-Cas9 (#50661) (Addgene, Watertown, MA, USA) and selected by puromycin. Next, gRNA was introduced by a separate lentiviral expression vector (#52963) (Addgene, Watertown, MA, USA). The seed region of gRNA against TGFBR1, TGFBR2 or non-targeting scramble (N/S) control are 5′-CATACAAACGGCCTATCTCG-3′, 5′-TGCTGGCGATACGCGTCCAC-3′, and 5′-ACGGAGGCTAAGCGTCGCAA-3′ respectively. To initiate Cas9-mediated gene knockout, the dual-lentiviral infected pools were pulse-treated with 500 ng/mL doxycycline for 3 days. TGF-β receptor knockout was subsequently confirmed by western blot and loss of TGF-β responsiveness.

### 2.9. Effect of TGF-β Pathway Inhibition on Chemotherapy Efficacy In Vivo

Xenografts were established in female SCID hairless mice (aged 4–5 weeks, Charles River Laboratories, Wilmington, MA, USA) (six mice per group) by intraperitoneal (i.p.) injection of 5 million SKOV3 N/S CRISPR cells or SKOV3 TGFBR2 CRISPR cells. Treatment was initiated 16 days after tumor inoculation by i.p. administration of cisplatin (2 mg/kg, every 6 days) or vehicle (normal saline). The mice were euthanized on Day 42 after tumor cell inoculation. Intraperitoneal tumor nodules (>1 mm diameter) were dissected and weighed to assess the tumor burden.

## 3. Results

### 3.1. Ovarian Cancer Cell Lines Demonstrated Constitutive and Inducible TGF-β Activity

**Figure 1:**
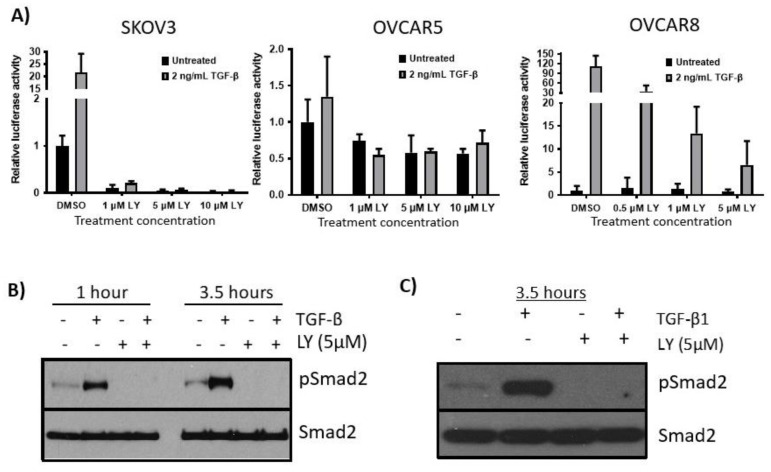
TGF-β signaling inhibition by LY2157299 in vitro. (**A**) Measuring SMAD4 transactivation activity via luciferase activity in SKOV3, OVCAR5, and OVCAR8 cells. Western blotting showing inhibition of Smad2 phosphorylation in ovarian cancer cells in (**B**) SKOV3 cells and (**C**) OVCAR8 cells. The uncropped blots are shown in Supplementary Materials.

A significant increase in TGF-β transcriptional activity (as measured by a TGF-β transcriptional luciferase reporter construct) was observed in SKOV3 cells upon exogenous TGF-β treatment (fold change = 21.73, *p* = 0.007). This activity was completely inhibited by 1 µM LY2157299 (Figure 1A). Given the near-complete inhibition seen at the lowest concentration of LY2157299, it is not surprising that higher concentrations had little additional impact.

In contrast, OVCAR5 cells were shown to be TGF-β unresponsive. TGF-β treatment resulted in a statistically insignificant increase in fold change between the TGF-β untreated and treated groups (fold change of 0.25; *p* = 0.4) (Figure 1A). This observation suggests that the TGF-β signaling integrity in these cells is not entirely intact.

OVCAR8 cells demonstrated an exquisite sensitivity to TGF-β, leading to a statistically significant increase in activity (fold change = 112.3, *p*-value = 0.003) (Figure 1A). This was fivefold more induction than SKOV3 and may reflect that SKOV3 cells have a baseline constitutive activation of the pathway. Comparing the effect of increasing doses of LY2157299 on the OVCAR8 cells treated with TGF-β, treatment with both 1 µM and 5 µM resulted in a dose-dependent inhibition of TGF-β transcriptional activity (88% decrease, *p*-value = 0.006 for 1 µM; 94.2% decrease, *p*-value = 0.005 for 5 µM).

Based upon these results, we determined that 5 μM is required to consistently inhibit the TGF-β treated ovarian cancer cell luciferase activity to a level comparable to or lower than the basal activity. This dose was used as the dosage in subsequent in vitro experiments for TGF-β signaling blockage. Consistent with this, Smad2 phosphorylation, a key step in TGF-β signaling, was completely inhibited by 5 µM LY2157299 in SKOV3 and OVCAR8 cells, as shown by western blotting (Figure 1B,C).

### 3.2. Ovarian Cancer Cell Lines Have a Broad Range of TGF-β Responsiveness

**Figure 2:**
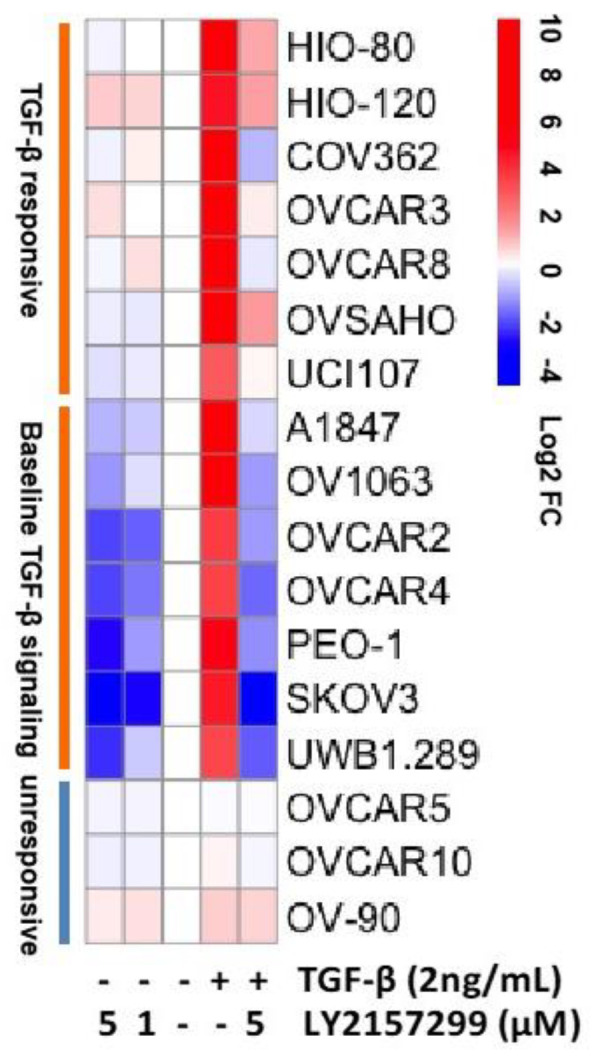
TGF-β responsiveness was screened via luciferase activity upon treatment with TGF-β (2 ng/mL) or TGF-βRI kinase inhibitor (LY2157299) (5 µM) for 24 hours in 17 ovarian cell lines (including 2 SV40 large T antigen immortalized ovarian surface epithelial cells). Log2 fold-change was plotted as a heatmap.

Given the different types of response to TGF-β treatment in these three cell lines, a broader screen of cell lines for TGF-β transcriptional response was assayed. To screen the TGF-β responsiveness of ovarian cancer, a CAGA12-luciferase assay using a Smad-binding element was applied to a panel of 15 human EOC cell lines treated with 2 ng/mL TGF-β and 1 or 5 µM LY2157299 [15]. The assay demonstrated statistically significant ligand-induced TGF-β signaling was prevalent in 12 (80%) cell lines, which can be effectively inhibited by 5 μM of LY2157299 (Figure 2). Furthermore, 7 (46.6%) of the EOC cell lines demonstrated some endogenous baseline TGF-β signaling. In these cell lines, reduction of baseline CAGA12-luciferase activity by LY2157299 was prominent, suggesting TGF-β signaling is activated in a subset of EOC via an autocrine loop. The cell lines were arranged into a heatmap by Log2 fold-change of luciferase activity grouped according to their fold change and TGF-β response: unresponsive (no response to TGF-β and no effect by LY2157299), constitutive signaling (endogenous TGF-β activity and susceptible to LY2157299), and inducible TGF-β activity only (responsive to TGF-β and susceptible to LY215799). These groups likely reflect the real spectrum of TGF-β effects on ovarian cancer, and the constitutively active cell lines may include those tumors which are difficult to debulk and treat.

### 3.3. Inhibition of the TGF-β Pathway Decreases the Migration and Invasion of Ovarian Cancer Cells

**Figure 3:**
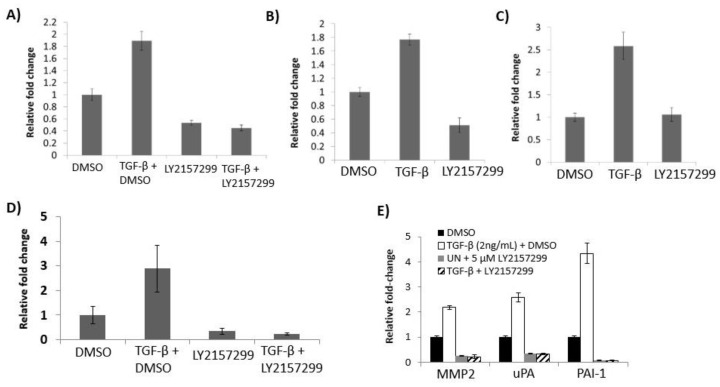
LY2157299 inhibits the migration of ovarian cancer cells. Ovarian cancer cells were allowed to migrate through a Boyden chamber overnight under a chemotaxis of 0.5% FBS (SKOV3) or 4% FBS. TGF-β (2 ng/mL) and LY2157299 (5 μM) were added to both the upper and lower chambers. (**A**) SKOV3; (**B**) OVCAR4; and (**C**) OVCAR8. (**D**) LY2157299 inhibits the invasion of SKOV3 cells through a Matrigel-coated Boyden chamber (0 to 2% FBS, overnight). (**E**) Measuring the fold change of genes (MMP2, uPA/PLAU, PAL-1) from the “debulking signature” that mediate tumor invasion with TGF-β and LY2157299 treatment.

To determine the role of TGF-β in ovarian cancer biology, the cellular migration of SKOV3, OVCAR4, and OVCAR8 cells was measured in a Boyden chamber-based migration assay in the presence or absence of TGF-β. TGF-β treatment was found to stimulate the migration of all three cell lines, with an average fold change of 2 (*p* = 6.96 × 10^5^, 0.002, and 0.001 respectively) (Figure 3A–C). The TGF-β inhibitor LY2157299 completely blocked the TGF-β-induced migration in SKOV3 and OVCAR4 cells (Figure 3A,B). More importantly, LY2157299 also downregulated the baseline migration of SKOV3 and OVCAR4 cells, suggesting that these cell lines have cellular motility based upon the baseline biochemical activation of the pathway (indicated by the decrease in relative luciferase activity upon treatment with LY2157299 alone), as demonstrated in Figure 1. In contrast, OVCAR8 showed no statistically significant differences between the untreated and LY2157299+TGF-β groups (Figure 3C). This indicates that only the TGF-β-induced cellular migration in OVCAR8 is blocked by LY2157299 treatment.

Given the TGF-β responsiveness of SKOV3 cells to cellular migration, we chose this cell line to examine the effects of TGF-β on cellular invasion. For the invasion assay, treatment with TGF-β led to an increase in SKOV3 cell invasion (fold change = 2.88; *p* = 0.032) (Figure 3D). SKOV3 TGF-β–induced invasion was inhibited by LY2157299 treatment (fold change = 0.22, *p* = 0.01). Moreover, the baseline invasive activity was also inhibited, similarly to migration (fold change = 0.34, *p* = 0.039) (Figure 3D). Three previously identified TGF-β regulated genes involved in tumor invasion (MMP2, uPA/PLAU, PAI-1) from the “debulking signature” for ovarian cancer were tested via qRT-PCR in SKOV3 cells treated with combinations of TGF-β and LY2157299 [8]. For all three genes, TGF-β treatment upregulated gene expression (fold change = 2, 2.5, 4.5, respectively). Treatment with LY2157299, however, led to downregulation of all three genes with fold changes below the untreated baseline, both alone and with TGF-β (Figure 3E).

### 3.4. LY2157299 Inhibits Adhesion of Ovarian Cancer Cells and Mesothelial Cells

**Figure 4:**
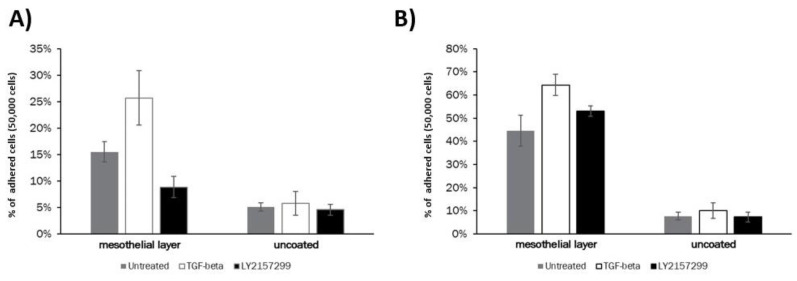
Cell adhesion assay with pretreated ovarian cancer cells. LY2157299 inhibits the adhesion of (**A**) SKOV3 and (**B**) OVCAR8 cells to a confluent mesothelial monolayer in vitro. Before culturing SKOV3 and OVCAR8 cells with a confluent monolayer of HM-3 cells, ovarian cancer cells were pretreated with TGF-β (2 ng/mL) and LY2157299 (5 μM) overnight.

For ovarian cancer cells to metastasize, they need to not only spread throughout the abdomen by detaching and migrating, but also by adhering to the peritoneal and bowel surfaces. Thus, cellular adhesion is a critical element in the anatomic spread of ovarian cancer. To determine the impact of TGF-β on ovarian cancer-associated mesothelial cell interactions, an assay was designed to measure the ability of TGF-β and LY2157299 pre-treated ovarian cancer cells to attach to a confluent monolayer of mesothelial cells. The TGF-β pretreated SKOV3 and OVCAR8 cells showed a 1.66-fold and 1.44-fold increase in adhesion activity compared to the untreated cells (*p* = 0.031 and 0.013, respectively), suggesting pro-adhesion effects of TGF-β signaling activation (Figure 4). Conversely, elevation of cellular adhesion activity was not observed in SKOV3 and OVCAR8 cells pretreated with TGF-β and LY2157299 (FC: −0.57 and 1.18, *p*-value: 0.014 and 0.107). Furthermore, the significant *p*-values underlying the decreased fold-change in SKOV3 indicate that the baseline adhesion activity was inhibited, which is consistent with the fact that SKOV3 possesses endogenous TGF-β activity. For the control, the pretreated SKOV3 and OVCAR8 cells were cultured alone on a dish without HM-3 (human mesothelial cells), and there was little change in the percentage of adhered cells between the treatment groups.

**Figure 5:**
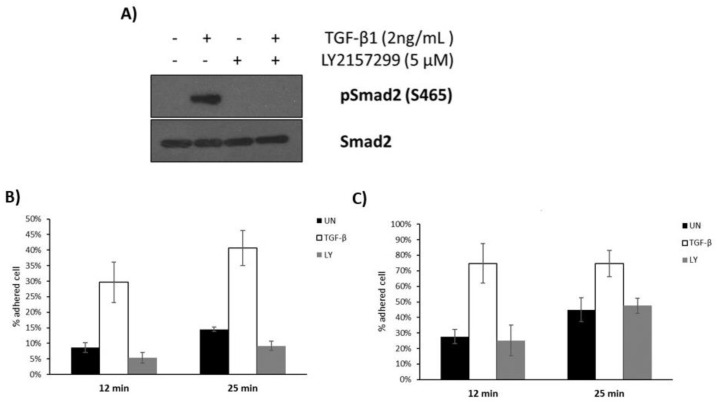
(**A**) Inhibition of Smad2 phosphorylation with LY2157299 in mesothelial cells (HM3). (**B**) SKOV3 and (**C**) OVCAR8 ovarian cancer cell adhesion assay (at 12 and 25 min incubations) with pretreated mesothelial cells and TGF-β (2 ng/mL) and LY2157299 (5 μM). The uncropped blots are shown in Supplementary Materials.

Considering that cell adhesion is a reciprocal process, the modulatory effect of TGF-β signaling in mesothelial cells was tested for their capacity as substrates for ovarian cancer adhesion. Phosphorylation of Smad2, an important step in TGF-β pathway activation, was shown to be upregulated in western blotting by TGF-β treatment and inhibited by LY2157299 treatment in HM-3 cultures (Figure 5A), demonstrating the presence of functional TGF-β signaling in HM-3 cells. An adhesion assay was performed using untreated ovarian cancer cells and HM-3 monolayers pretreated with TGF-β with or without LY2157299. Pretreating HM-3 cells with TGF-β alone resulted in an increase in adhesion over time (a fold change of around 3 for 12 min and 4 for 25 min for SKOV3, and a fold change of around 3 for 12 min and 2 for 25 min for OVCAR8), which was blocked by LY2157299 regardless of the ovarian cancer cell line (SKOV3 or OVCAR8) (Figure 5B,C). Taken together, our observation demonstrated the involvement of TGF-β in the interaction between ovarian cancer cells and peritoneum mesothelial cells, which resulted in increased adhesion, a prerequisite for the peritoneal dissemination of cancer.

### 3.5. Cancer-Associated and Normal Fibroblast-Stimulated Ovarian Cancer Cell Proliferation Is TGF-β Dependent

**Figure 6:**
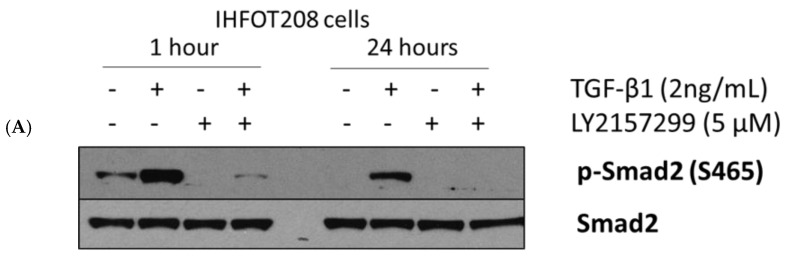

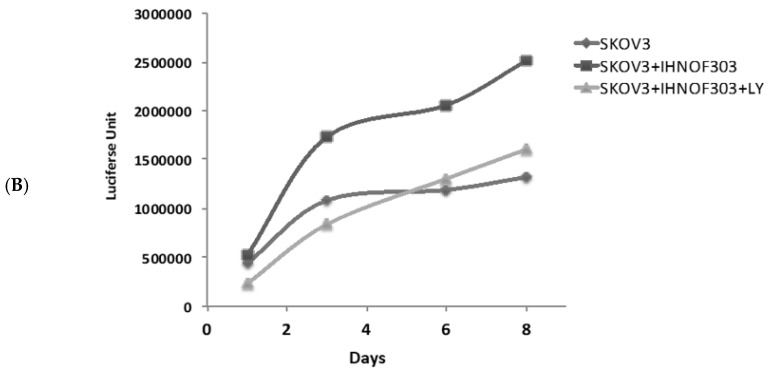
(**A**) Inhibition of Smad2 phosphorylation in cancer-associated fibroblasts (IHFOT208) after incubation with TGF-β and LY2157299 at 1 and 24 h. (**B**) Luciferase assay measuring growth of SKOV3 cells in a 1:1 co-culture with normal fibroblasts (IHNOF303) over 8 days. The uncropped blots are shown in Supplementary Materials.

Cancer-associated fibroblasts are a critical component of ovarian cancer that support tumorigenesis [17]. Cancer-associated fibroblasts (IHFOT208) treated with TGF-β for 1 and 24 h demonstrated increased phosphorylation of Smad2 by western blotting, which was inhibited by treatment with LY2157299 (Figure 6A). To test the impact of fibroblast co-culture on the proliferation of ovarian cancer cells, a stable luciferase-overexpressing SKOV3 cell line (SKOV3-Luc) and normal fibroblasts (IHNOF303) were 1:1 mixed and seeded in a 96-well plate. As shown in Figure 6B, SKOV3 co-cultured with IHNOF303 showed an average of 2-fold increase in proliferation (*p* = 0.003), demonstrating that fibroblasts stimulate the growth of SKOV3 ovarian cancer cells. Treatment with LY2157299 significantly inhibited the proliferation of co-cultured SKOV3-Luc cells, showing that the IHNOF303 cells have TGF-β activity and enhance SKOV3 cell growth.

### 3.6. TGF-β Signaling Inhibition by CRISPR-Mediated Knockout of TGFBR1 and 2 in Ovarian Cancer Cells

**Figure 7:**
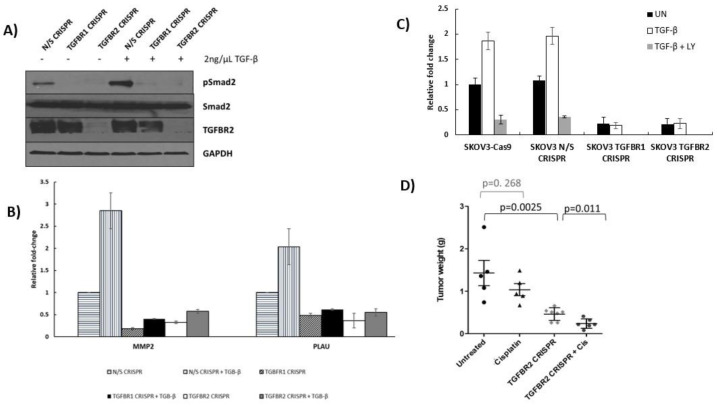
Impact of *TGF-β* pathway induction and inhibition in SKOV3 TGFBR1 and 2 CRISPR cells. (**A**) Western blotting of pSmad2, Smad2, TGFBR2, and GADPH in TGF-β-treated TGFBR1 and TGFBR2 CRISPR SKOV3 cells. (**B**) qRT-PCR of MMP2 and uPA/PLAU expression in SKOV3 cells with and without TGF-β inhibition. (**C**) SKOV3 cell proliferation compared and measured as the relative fold change between the N/S CRISPR cells and the TGFBR1 and TGFBR2 CRISPR cells upon treatment with TGF-β and LY2157299. (**D**) In vivo anti-tumor efficacy and chemotherapy sensitization of TGF-β inhibition. Post-dissection tumor weight of SKOV3 cells treated with i.p. cisplatin, and TGFBR2 CRISPR SKOV3 cells treated with i.p. cisplatin. The uncropped blots are shown in Supplementary Materials.

To independently validate the effects of LY2157299 in the in vitro model as an on-target effect, the impact of TGF-β signaling inhibition by CRISPR-mediated knockout of the TGF-β receptors on ovarian cancer biology was tested. CRISPR knockout of TGFBR1 and TGFBR2 in separate SKOV3 cell lines demonstrated a complete blockage of pSmad2, both untreated and with TGF-β induction, as shown by western blot analysis (Figure 7A). Furthermore, the mRNA expression of MMP2 and uPA/PLAU induced by endogenous or exogenous TGF-β was completely inhibited in SKOV3 TGFBR1 KO and SKOV3 TGFBR2 KO cells. As shown in Figure 7B by qRT-PCR, MMP2 and PLAU showed approximately 2.5- and 1.75-fold change, respectively, upon TGF-β treatment in SKOV3 N/S CRISPR cells (scramble gRNA control). Conversely, a dramatic downregulation of gene expression occurred with knockout of TGFBR1, with expression falling well below the baseline for both genes (relative fold changes of around 0.2) in cells without TGF-β treatment. Addition of TGF-β did not lead to upregulation of gene expression in the TGFBR1 CRISPR cells, with the relative fold change remaining close to that of cells without TGF-β (Figure 7B). A similar effect was noted in the SKOV3 TGFBR2 CRISPR cells, with the relative fold change for the TGF-β untreated cells slightly above 0, and the relative fold change for the TGF-β treated TGFBR2 CRISPR cells at similar levels to that of the TGF-β treated TGFBR1 CRISPR cells.

The TGF-β receptor knockout cell lines provided ideal models to independently test the role of TGF-β on ovarian cancer cell migration. The SKOV3 CRISPR cell lines were subjected to the same migration assay as described in Figure 3A. Cas-9 overexpressing SKOV3 cells or the scramble control SKOV3 CRISPR cells showed similar behavior as wild-type SKOV3 cells in response to TGF-β or TGF-β with added LY2157299 treatments (Figure 7C). In contrast, the migration activity associated with either endogenous TGF-β signaling activity or exogenous TGF-β treatment was blocked by CRISPR knockout of TGFBR1 or TGBFR2. These observations demonstrate that receptor knockout has a similar effect to LY2157299 treatment for TGF-β signaling inhibition.

### 3.7. TGF-β Pathway Inhibition Enhances Anti-Tumor Efficacy and Chemotherapy Sensitivity in an In Vivo Mouse Model

SKOV3 cells with CRISPR-mediated TGFBR2 knockout were tested for in vivo tumor growth and sensitivity to chemotherapy. As TGFBR2 is the ligand-binding cell surface receptor that phosphorylates TGFBR1 and begins the TGF-β signaling cascade, CRISPR knockout of this receptor would inhibit the entire signaling pathway upstream of all signaling in the cells. Like what was shown in Figure 7C, inhibition of TGF-β signaling by TGFBR2 knockout showed an approximately 67% decrease in tumor weight (*p* = 0.0025) (Figure 7D). Moreover, TGF-β inhibition increased the susceptibility of SKOV3 cells to cisplatin treatment. For the treatment regimen, cisplatin alone did not result in a statistically significant decrease in tumor burden (*p* = 0.268) in wild-type SKOV3 cells due to the SKOV3 cell line being platinum resistant [18,19,20]. In contrast, TGFBR2 CRISPR SKOV3 cells showed a statistically significant tumor weight decrease upon the same cisplatin treatment (52% tumor weight decrease) compared to untreated TGFBR2 CRISPR SKOV3 cells (*p* = 0.011). This adds to the evidence that TGF-β pathway inhibition has an additive anti-tumor effect with chemotherapy and could increase chemotherapy response in EOC.

## 4. Discussion

The need for a surgical approach to advanced-stage ovarian cancer has not measurably changed over the last 60 years, with the exception of the demonstration that interval debulking is equally effective as upfront debulking. Multiplerandomized studies have demonstrated that surgery at either point is effective [21,22,23,24,25]. However, one constant element is that suboptimally debulked ovarian cancer patients have a significantly shortened progression-free and overall survival. The prognostic impact of suboptimal debulking had never been understood from a mechanistic standpoint. Clearly, surgical ability is critical to this issue, as poor surgical skills will certainly contribute to a suboptimal debulking outcome. However, there is an equally likely biological basis for suboptimal debulking status. Our previous work identified a gene expression signature that predicts suboptimal debulking results [9]. This signature was centered on the TGF-beta pathway, strongly suggesting that the activation of this pathway makes ovarian cancers difficult to fully resect. It is obvious that a predictive tool to identify patients with suboptimally debulkable tumors ahead of surgery would be extremely valuable. Recent developments have shown that debulking surgery done initially or later makes essentially no difference in patient survival if a complete resection is accomplished [24,26]. In the study by Kobal et al. (2018) [24], the researchers compared the overall survival (OS) and progression-free survival (PFS) of patients who underwent primary debulking vs. NACT-IDS based on the post-operative residual disease. The study found that, while the post-surgery macroscopic residual disease and the rate of post-operative complications were lower in NACT-IDS patients compared to the PDS patients, there was no statistically significant difference in OS and PFS between the groups [24]. However, in the study by Gill et al. (2017) [26], a significant difference in OS was seen in patients undergoing NACT-IDS compared to patients who underwent primary debulking, while the PFS was not significant between the compared groups. The study also showed that NACT-IDS can benefit a subset of patients, particularly those at the highest risk of suboptimal debulking or patients with the highest risk of surgical morbidity and adverse outcomes [26].

**Figure 8:**
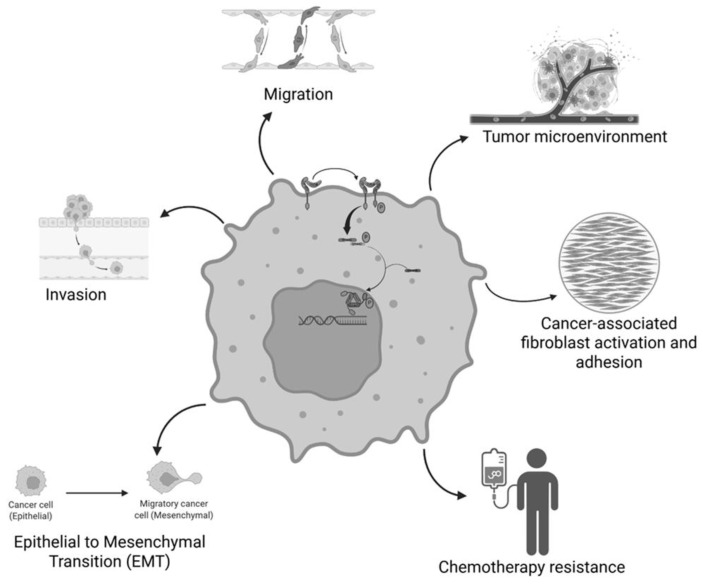
Cancer biology impacted by TGF-β pathway activation.

However, the real challenge is understanding the biology behind the signature. This manuscript describes a series of important experiments to elucidate the biology of the TGF-beta pathway in ovarian cancer. In order to dissect the TGF-beta pathway and its role in ovarian cancer biology, an effective method of manipulation of the pathway is critical. The TGF-βR1 small molecule inhibitor LY2157299 fit these requirements and was used to determine the effects of TGF-β inhibition on ovarian cancer features that might be relevant to cancer biology consistent with suboptimally debulked tumors (Figure 8). These features would include tumor migration, invasion and adhesion, which are essential for metastasis of ovarian cancer and disseminated disease that might contribute to clinical features, which would contribute to suboptimal disease. This study demonstrates that treatment with TGF-β upregulated both cellular migration and invasion in the ovarian cancer cell lines SKOV3 and OVCAR8. LY2157299 treatment decreased both processes, even with exogenous TGF-β treatment in culture. The prevention of tumor cell migration and invasion is very relevant for patients more likely to be suboptimally debulked.

Along with the biologic effects directly on ovarian cancer cells, the impact of TGF-β pathway inhibition on other tissues is important for the progression of ovarian cancer. The TGF-β pathway clearly plays a major role in the adhesion of ovarian cancer cells, both through direct effects on the cancer cells and by priming the mesothelial lining of the peritoneum. In addition, our results demonstrate a role for TGF-β in the tumor microenvironment. The tumor microenvironment is thought to play a role in metastasis, immunosuppression, angiogenesis, EMT, and extracellular matrix remodeling, along with shaping tumor behavior and crosstalk between the tumor and the microenvironment [27,28]. Tumor-associated fibroblast proliferation was stimulated by ovarian cancer cells (presumably by secreted factors), and this was blocked with LY2157299 treatment, strongly suggesting that the TGF-β pathway was critical in mediating this effect. It is important to note that the tumor microenvironment is made up of diploid cells, as are mesothelial cells, all of which are genomically stable and thus make them especially good therapeutic targets. Further experiments will need to be conducted to determine the effectiveness of TGF-β inhibiting agents on ovarian cancer and to determine potential toxicities in normal tissues.

While this study demonstrates that inhibition of TGF-β may be a reasonable therapeutic intervention for ovarian cancer, LY2157299 is unlikely to be that agent. We utilized a murine cancer model with SKOV3 ovarian cancer cells treated with LY2157299, and the results indicated that there is not a statistically significant difference between tumor weights in treated versus untreated subjects. This is likely the result of the insolubility of LY2157299, not the efficacy of the drug itself. The drug could not be adequately suspended in PBS and required a vehicle of 20% DMSO, 0.5% carbosymethylcellulose, and 0.5% Tween-80 for injection. There was no indication post-intraperitoneal injection that the treatment distributed throughout the peritoneal cavity to treat the tumors, and it may have precipitated in the animals’ tissues. Other investigators have had reasonable success using LY2157299 with an intraperitoneal injection treatment model, such as Zhang et al. (2018) [29]; however, their model differed from our described murine model [29]. Their model relied upon pretreatment with LY2157299 for 2 days prior to xenograft formation and treated for four weeks, twice daily, seven days a week. A treatment course such as this can allow for a large concentration of the drug to build up in the tissues of the mice over a larger time frame and lead to greater effectiveness than was seen in our study. However, this schedule is not clinically viable, as patients are unable to be treated prior to tumor introduction in most cases. They are also unable to be treated twice daily every single day during their treatment course, with most chemotherapy courses involving intravenous drug infusion over the course of hours and potentially multiple times weekly or monthly, but not such intensive schedules.

As shown in Table 1, we have contrasted the previously mentioned study with two other studies involving LY2157299 in ovarian cancer. While Zhang et al. (2018) [29] showed in vivo effectiveness of the molecule, other studies only investigated the effectiveness of LY2157299 in vitro and as an alternative treatment modality to radiotherapy and sorafenib (a pankinase inhibitor) (Tian et al. [2022]) and determining the NK cell-suppressive ability of TGF-β1 and the ability of LY2157299 to restore NK cell function (Maas et al. [2024]) [30,31]. While these studies determined some of the behaviors we tested (migration and invasive ability of the cell lines), we also determined the impact of TGF-β pathway inhibition on Smad2 phosphorylation (a key component in activation of the TGF-β pathway) and mesothelial cell-mediated cell-to-cell adhesion, that of ovarian cancer cell to mesothelial cell, which is important in the formation of the tumor microenvironment and many of the tumor behaviors that present in high-grade ovarian cancer. Our study provided these novel insights using LY2157299 as the main treatment modality, indicating that, while the molecule may not have validity as a single target agent in the clinical setting, it has validity as a helpful laboratory testing method of TGF-β pathway inhibition.

As stated, our study not showing significant in vivo results does not preclude TGF-β as a treatment target in ovarian cancer, with our data showing that inhibition of TGF-β impacts multiple tumor cell lines and other microenvironment and peritoneal cell types. Over the last several years, multiple TGF-β inhibitors have entered the clinic, and some have been FDA approved (diabetic nephropathy) [32,33]. Several of these are neutralizing antibodies, which could be very effective, especially in combination with chemotherapy. Platinum-based chemotherapy, often in combination with taxane chemotherapies, is the standard of care treatment for ovarian cancer [6]. It has been suggested that chemotherapeutic resistance can be a result of tumor microenvironment changes mediated by TGF-β and, as such, would need to be analyzed in vivo to determine the effect of TGF-β pathway inhibition [18].

The current findings provide the translational rationale for future clinical and mechanistic investigations. The objective of the present study was to establish the clinical relevance of TGF-β pathway inhibition in ovarian cancer treatment response rather than to define its underlying molecular mechanisms. These results provide an important translational bridge between our observations and future experimental studies evaluating the molecular interactions between TGF-β inhibition and chemotherapy response in both in vitro and in vivo ovarian cancer models, along with future directions for clinical studies.

This is particularly important as our data did show synergism between TGF-β inhibition and chemotherapy, as shown in our CRISPr experiments. Recently, the laboratory has published a high-profile study identifying proteogenomic signatures which predict for ovarian refractoriness, and a subset of these patients have a TGF-β gene signature [8]. This data strongly supports the role of the TGF-β pathway in both the early development and progression of epithelial ovarian cancer.

## 5. Conclusions

In the in vitro experiments conducted, TGF-β pathway inhibition successfully targeted many of the ovarian cancer cell behaviors that could contribute to suboptimally debulked patients and showed an additive anti-tumor effect in conjunction with cisplatin. LY2157299 treatment is clinically limited due to solubility, as previously mentioned, but other TGF-β inhibitors could be highly efficacious treatment options. Future studies will test the efficacy of other small molecule inhibitors or monoclonal antibodies in conjunction with chemotherapy to target the ovarian cancer behaviors that contribute to suboptimal debulking status and determine the validity of TGF-β inhibition as a therapy for ovarian cancer.

## Figures and Tables

**Table 1:** Comparison of previous ovarian cancer studies using LY2157299 in in vivo and in vitro assays.

Citation	Cell Lines Used	Assays Used	Use of LY2157299 in Study	Investigated Impact on TGB-β Pathway
Zhang, et al., 2018 [29]	OVCAR8, CAOV3	Cell proliferation, clonogenic assay, wound healing scratch assay, Matrigel cell invasion, microarray and real-time qPCR, Western blot, i.p. xenograft OC model, IHC	In vitro and in vivo studies with LY2157299 inhibiting TGB-β signaling and cancer-stroma crosstalk, main drivers of ovarian cancer function and the tumor microenvironment	The TGB-β pathway activity is modulated with LY2157299 through in vitro analysis of cells and tumor stroma (namely TGB-β1 activation of CAFs), along with an in vivo study of tumor growth and ascites development in an ovarian cancer mouse model
Tian, et al., 2022 [30]	SKOV3	Cell viability, transwell migration, tumor cell wound healing scratch assay, radiosensitivity assays	LY2157299 was used to block the TGB-β1-mediated ovarian cancer activities in vitro (i.e. EMT, migration, radioresistance)	Sorafenib (a pankinase inhibitor) was used to regulate TGB-β1-mediated tumor cell proliferation, mobility, and radiosensitivity, and LY2157299 was used as a comparable treatment
Maas, et al., 2024 [31]	SKOV3 patient ascites-derived NK cells and EOC cells	Luminex, ELISA, flow cytometry, NK cell functionality assays	Treatment of NK cells with LY2157299 to restore proliferative potential and how this restores the functionality of NK cells	TGB-β and NK cell interations in EOC patient ascites, determining the NK cell-suppressive ability of TGB-β1

## Data Availability

The original contributions presented in this study are included in the article/Supplementary Materials. Further inquiries can be directed to the corresponding authors.

## Supplementary Materials

The following supporting information can be downloaded at: https://www.mdpi.com/article/10.3390/cancers18142268/s1, Original Western Blot.
