# Supplementary material for: TGF-β Is Critical for Ovarian Cancer Migration, Invasion, and Chemosensitivity
Source: Cancers (Basel). 2026 Jul 15;18(14):2268. doi: 10.3390/cancers18142268 (PMC13406158; doi:10.3390/cancers18142268)
Supplement: Supplementary file 1 [file cancers-18-02268-s001.zip › Original Western Blot Scans.pptx]

## Slide 1
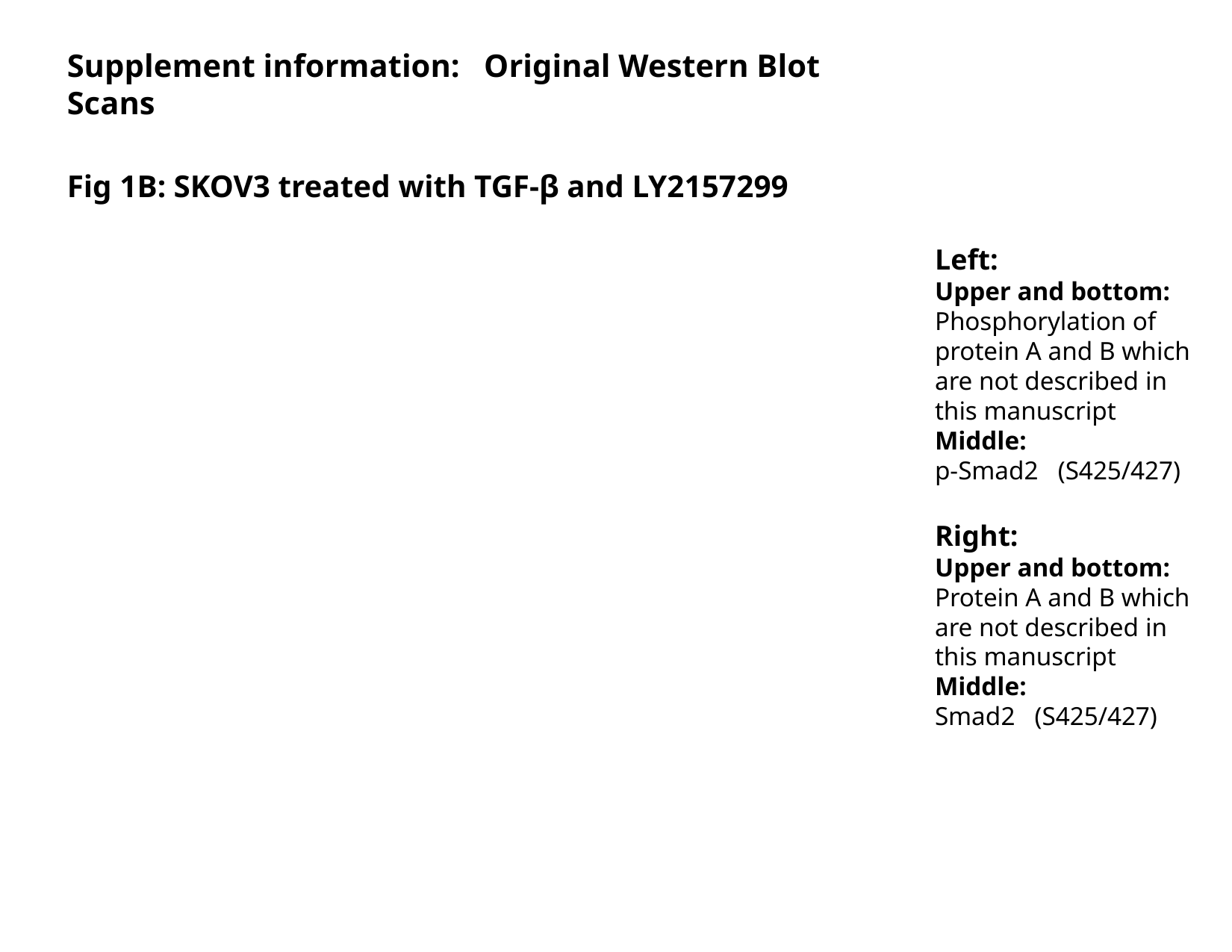

Supplement information: Original Western Blot Scans
Fig 1B: SKOV3 treated with TGF-β and LY2157299
Left:
Upper and bottom:
Phosphorylation of protein A and B which are not described in this manuscript
Middle:
p-Smad2 (S425/427)
Right:
Upper and bottom:
Protein A and B which are not described in this manuscript
Middle:
Smad2 (S425/427)

## Slide 2
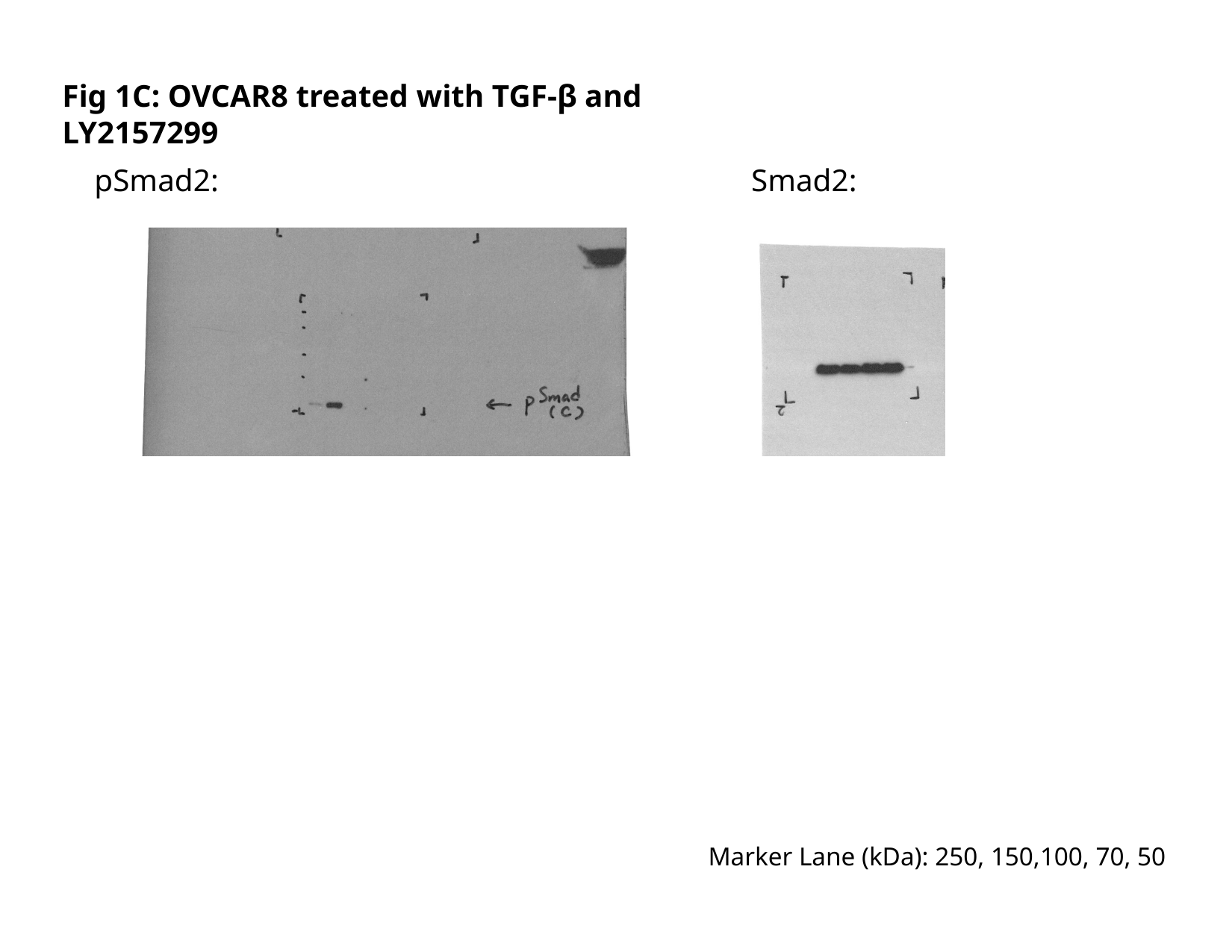

Fig 1C: OVCAR8 treated with TGF-β and LY2157299
pSmad2:
Smad2:
Marker Lane (kDa): 250, 150,100, 70, 50

## Slide 3
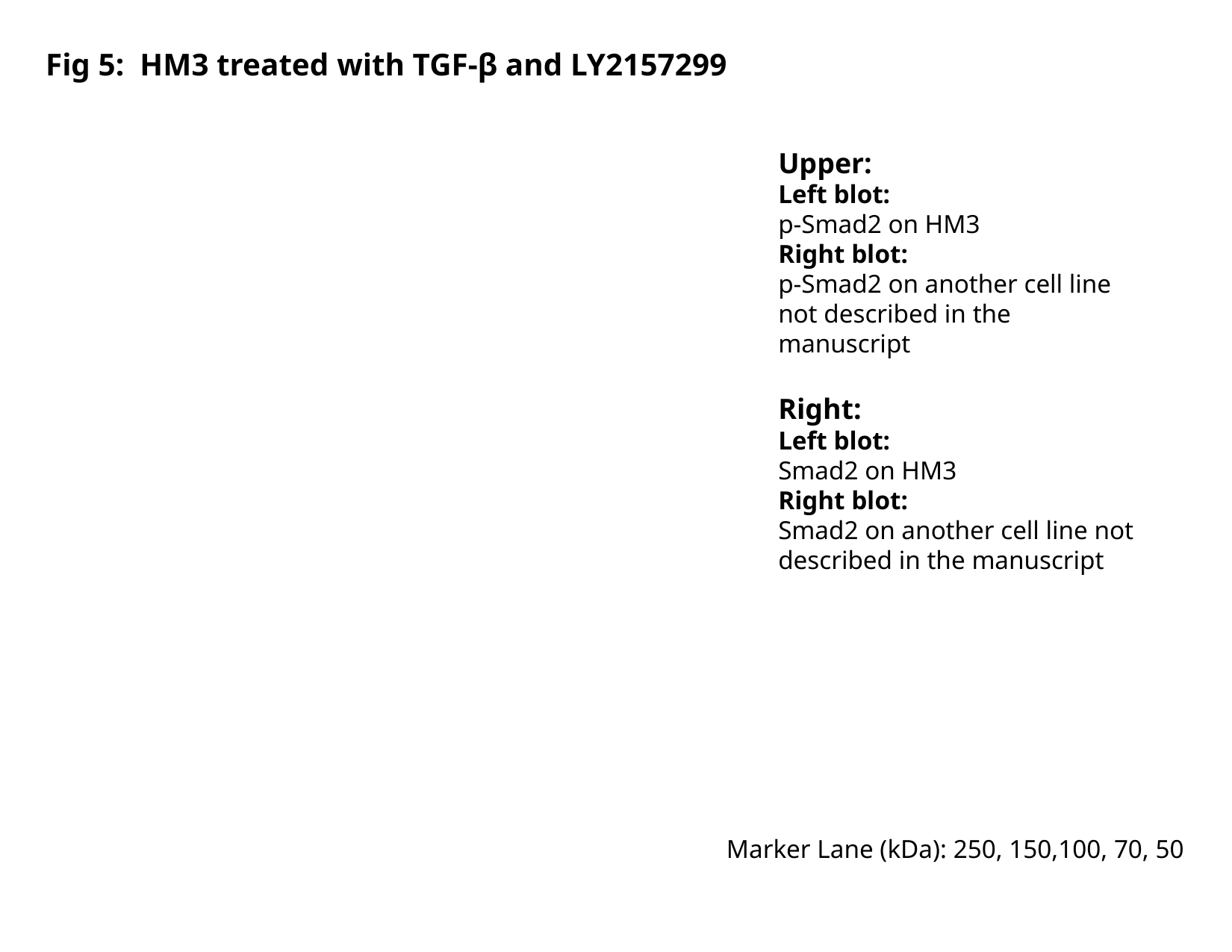

Fig 5: HM3 treated with TGF-β and LY2157299
Upper:
Left blot:
p-Smad2 on HM3
Right blot:
p-Smad2 on another cell line not described in the manuscript
Right:
Left blot:
Smad2 on HM3
Right blot:
Smad2 on another cell line not described in the manuscript
Marker Lane (kDa): 250, 150,100, 70, 50

## Slide 4
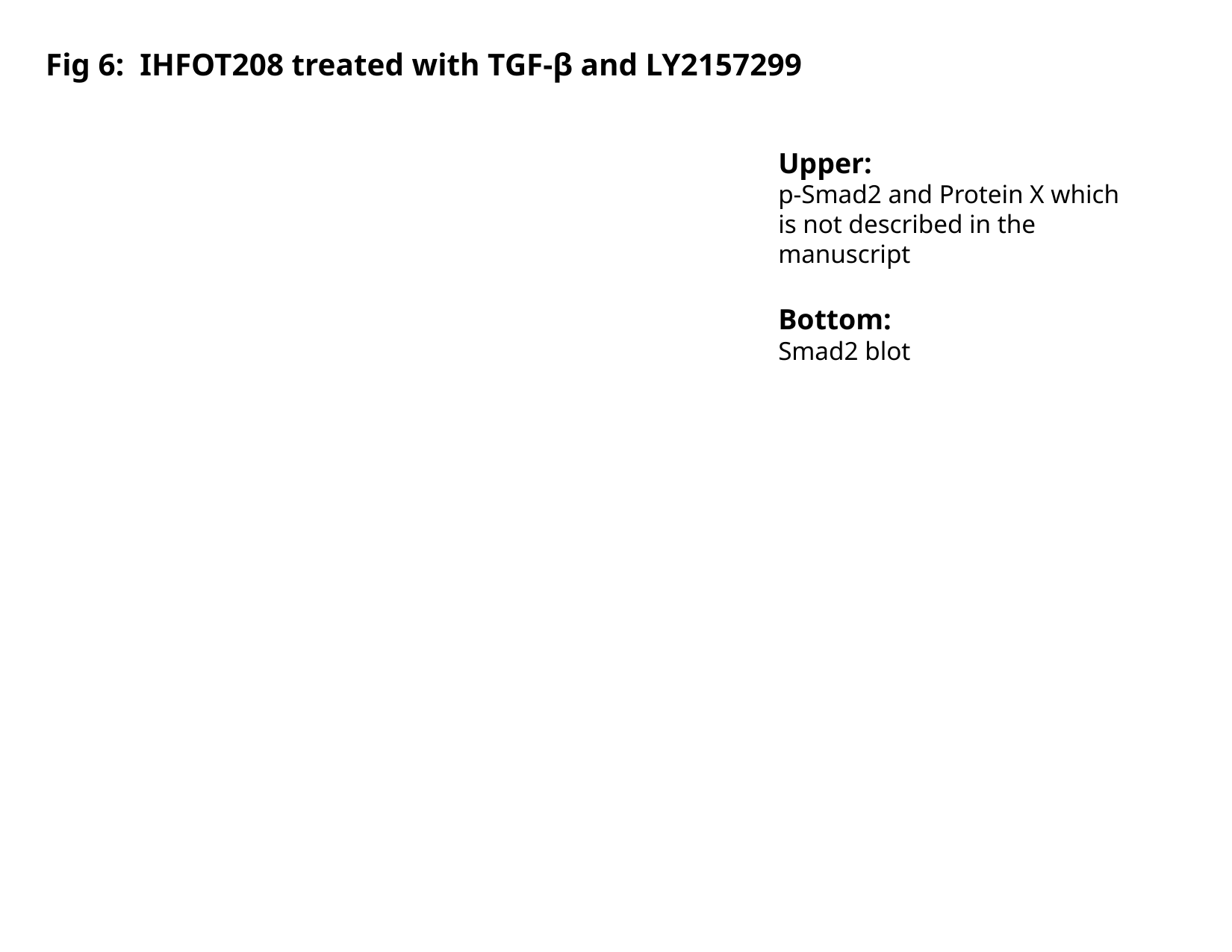

Fig 6: IHFOT208 treated with TGF-β and LY2157299
Upper:
p-Smad2 and Protein X which is not described in the manuscript
Bottom:
Smad2 blot

## Slide 5
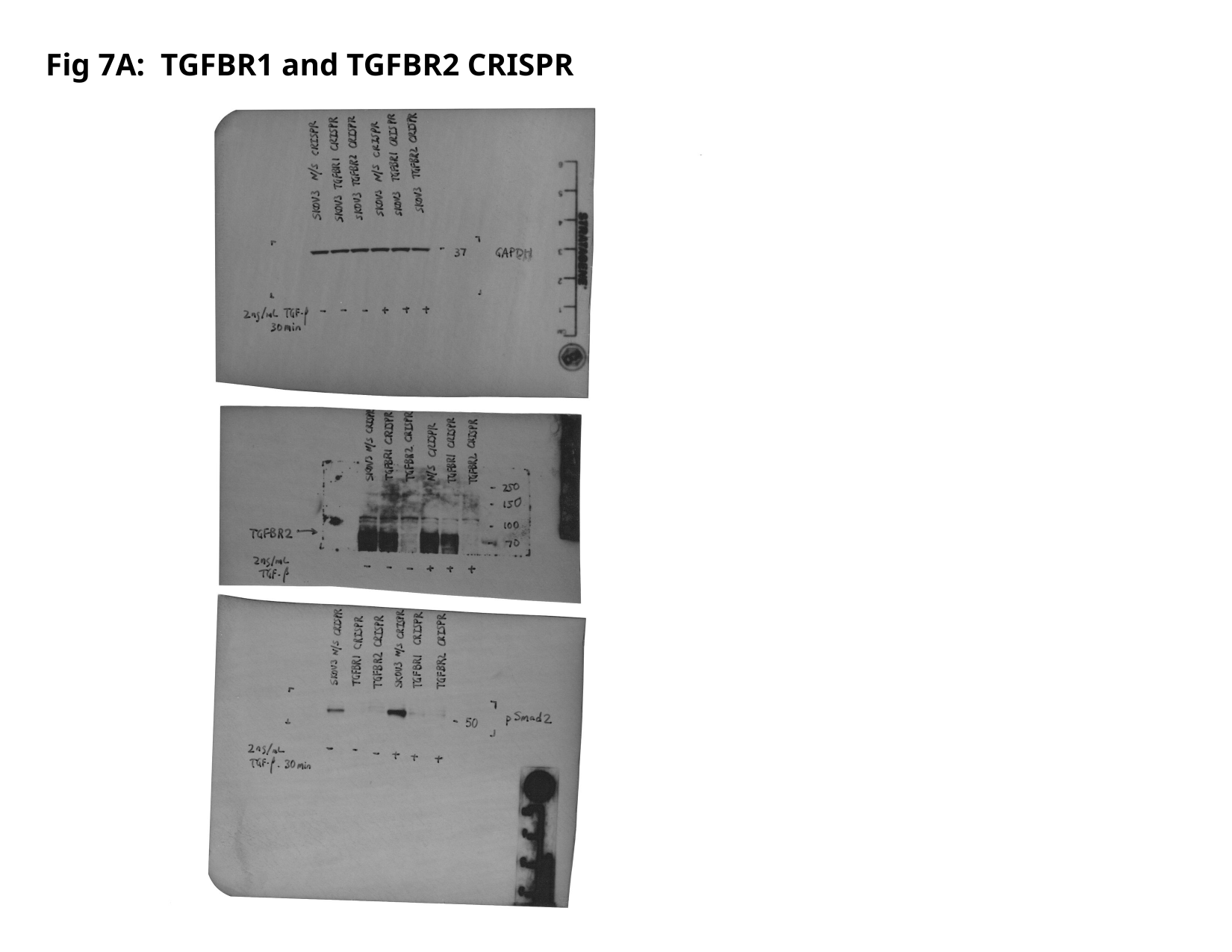

Fig 7A: TGFBR1 and TGFBR2 CRISPR
